# A daunting task for PAHO's new leadership

**DOI:** 10.1016/j.lana.2022.100379

**Published:** 2022-10-14

**Authors:** The Lancet Regional Health– Americas

The Pan American Health Organization (PAHO) was created in December, 1902, in response to the yellow fever epidemic that killed more than 20 000 people in the Americas. Over its 120 years of existence, PAHO has evolved from a body that defines procedures and guidelines to hamper the spread of a disease, to one that occupies an essential role in supporting and advancing the entire regional health agenda. Over the past 3 years of the COVID-19 pandemic, the region of the Americas has been the hardest hit with 2.8 million lives lost, accounting for 43% of the global death toll. According to PAHO's annual 2022 Health in the Americas report, life expectancy fell by 2·9 years in Latin America and the Caribbean and by 1·8 years in North America. Major interruptions in essential health-care services reversed years of advances in prevention and treatment of communicable and non-communicable diseases, and vaccination programmes. Between 2021 and 2022, child vaccination coverage has fallen below 80% in nearly all South American countries, which are now at serious risk of measles and polio outbreaks.

At the 30th Pan American Sanitary Conference that took place in September 2022, health ministers and leaders from PAHO member states congregated to discuss the future of the region and elect PAHO's new director, who will lead the organisation for the next 5 years and undertake the daunting responsibility to guide the region through recovery. On the conference's agenda, the focus was on increasing genomic surveillance, strengthening human resources for health, and advancing universal health care. In her opening speech, PAHO's former director, Carissa Etienne reminded us that “disease knows no borders” and that we need to strengthen genomic surveillance and commit to collecting and sharing epidemiological data. In 2020, the COVID-19 Genomic Surveillance Regional Network (COVIGEN) was created to support the identification and tracking of new variants within the region, and now it could be extended to collect genomic data for the region—including data from the ongoing monkeypox outbreak—to support local health authorities and researchers. PAHO's outstanding record in supporting technical cooperation between countries and partnering with ministers of health, civil societies, and other international organisations speaks positively about how COVIGEN can grow into a reference tool to improve the region's emergency preparedness and help prevent future major outbreaks.

The health-care workforce has suffered disproportionately during the COVID-19 pandemic, so recognising the need to expand and invest in the training of health-care staff is paramount. Pointed out by authorities and representatives in the audience of the conference, women make up 70% of health-care workers and Black women are overrepresented in lower-paying health-care jobs; thus, when reforming our health-care system, it is imperative to account for gender and race and ethnicity disparities within this sector. During the conference, PAHO and the U.S. Department of Health & Human Services presented a plan to improve the availability and quality of health workers in the region. Their initiative, the Joint Action to Strengthen the Health Workforce in the Region of the Americas, will facilitate the training of 500 000 health-care professionals in the next 5 years, expand PAHO's virtual campus, and create a consortium of academic centres in public health. The initiative is expected to facilitate a broader plan adopted by the member states in June 2022, at the IX Summit of the Americas: The Action Plan on Health and Resilience in the Americas. The action plan pledges to strengthen training and education, increase public financing for health, and to expand equitable access to quality health services. Disparities in access to health care and structural inequities within the region were exposed and exacerbated by the current COVID-19 pandemic, with systematically marginalised and vulnerable populations bearing the highest burden of the disease. A study done in Brazil, a country with the second highest absolute number of deaths globally, showed that universal access to primary health care mitigated inequalities in COVID-19 vaccination among the most vulnerable populations. Advancing universal health care and fighting for health for all should be a priority for the region, and PAHO's leadership will be essential for moving this agenda further, so that action points become achievements.

As we recover once more from a devastating pandemic, PAHO is again to be a crucial part of our reconstruction plan. During the conference, PAHO elected its new director, Dr Jarbas Barbosa, who has inherited a fragmented region and a long list of challenges. Barbosa, the former assistant director, will lead the organisation through its mission to promote cooperation between countries to advance their health goals and improve the quality of the lives of the people of the Americas. At the same event, member states representatives and ministers of health from the region reaffirmed their commitments to meet the Sustainable Development Goals 2030 agenda. Getting back on track, and ensuring a sustainable recovery that leaves no one behind, will be a Herculean challenge for the Americas, and we will need everyone on board.

